# Peer Review of “Exploring the Reasons for Low Cataract Surgery Uptake Among Patients Detected in a Community Outreach Program in Cameroon: Focused Ethnographic Mixed Methods Study”

**DOI:** 10.2196/39106

**Published:** 2022-06-09

**Keywords:** ophthalmologic surgical procedures, access to health care, ophthalmology, patient-centered care, ethnography, health knowledge, attitudes, practice

*This is a peer-review report submitted for the paper “Exploring the Reasons for Low Cataract Surgery Uptake Among Patients Detected in a Community Outreach Program in Cameroon: Focused Ethnographic Mixed Methods Study.”*

## Round 1 Review

Dear Authors,

Thank you very much for this interesting paper [1] about cataract surgery uptake in Cameroon. I have nothing to add.

Kind regards!
